# Training primary healthcare workers in China’s township hospitals: a mixed methods study

**DOI:** 10.1186/s12875-020-01333-4

**Published:** 2020-12-02

**Authors:** Xuan Zhao, Haipeng Wang, Juan Li, Beibei Yuan

**Affiliations:** 1 China Center for Health Development Studies, Peking University, Beijing, China; 2 Centre for Health Management and Policy Research, School of Public Health, Cheeloo College of Medicine, Shandong University, Jinan, Shandong China; 3 Shandong Provincial Hospital, Jinan, Shandong China

**Keywords:** In-service training, Primary health workers, Job satisfaction, Competence, Performance, Mixed methods

## Abstract

**Background:**

Primary health care (PHC) was a keystone toward achieving universal health coverage and Sustainable Development Goals (SDGs). China has made efforts to strengthen its PHC institutions. As part of such efforts, regular in-service training is crucial for primary healthcare workers (PHWs) to strengthen their knowledge and keep their skills up to date.

**Objective:**

To investigate if and how the existing training arrangements influenced the competence and job satisfaction of PHWs in township hospitals (THs).

**Methods:**

A mixed method approach was employed. We analyzed the associations between in-service training and competence, as well as between in-service training and job satisfaction of PHWs using logistic regression. Interviews were recorded, transcribed, and analyzed using NVivo12 to better understand the trainings and the impacts on PHWs.

**Results:**

The study found that training was associated with competence for all the types of PHWs except nurses. The odds of higher competence for physicians who received long-term training were 3.60 (*p* < 0.01) and that of those who received both types of training was 2.40 (*p* < 0.01). PHWs who received short-term training had odds of higher competence significantly (OR = 1.710, *p* < 0.05). PHWs who received training were more satisfied than their untrained colleagues in general (OR = 1.638, *p* < 0.01). Specifically, physicians who received short-term training (OR = 1.916, *p* < 0.01) and who received both types of training (OR = 1.941, *p* < 0.05) had greater odds of general job satisfaction. The odds ratios (ORs) of general job satisfaction for nurses who received short-term training was 2.697 (*p* < 0.01), but this association was not significant for public health workers. The interview data supported these results, and revealed how training influenced competence and satisfaction.

**Conclusions:**

Considering existing evidence that competence and satisfaction serve as two major determinants of health workers’ performance, to further improve PHWs’ performance, it is necessary to provide sufficient training opportunities and improve the quality of training.

## Background

According to the Declaration of Alma-Ata [1], PHC is an essential element of the health system and is designed to provide universally accessible essential health care to individuals and families in the community as the first level of contact with the national health system. The performance of the PHC system is the key to and the foundation of the entire health system’s performance. The draft Astana declaration (Alma-Ata 2.0) restates the key principles of PHC and renews them as driving forces for achieving the SDGs [2, 3]. In China, THs constitute a critical part of the PHC delivery system by providing services ranging from basic public health services (such as preventive and health management care) to clinical outpatient and inpatient care for common diseases. In addition, they also take responsibility in supervising and providing technical guidance for the village clinics [4], the grassroots and first contact health care providers in rural China. The Chinese government launched a health care reform in 2009, reiterating the importance of PHC. As a result of the reforms aimed at strengthening the PHC system, the PHWs in THs who play a crucial role in improving the availability and quality of primary health services [5] have changed their role and increased their work responsibilities. However, like most of the low- and middle- income countries (LMICs), China is plagued by PHWs’ disappointing performance. Many studies confirmed that the existing PHWs performed poorly in both preventive and clinical services such as chronic disease management, tuberculosis diagnosis and treatment, as well as antibiotics prescription [6–8]. Health professionals’ performance is directly determined by both their competence and motivation [9]. Competence is associated with education background, in-service trainings, and experiences. The shortage of PHWs with enough education background is a long-standing issue in China: in 2010, 5.6% of health workers in THs had received formal medical undergraduate education (i.e. 5 years of medical education) (Ministry of Health, 2011). In 2017, the figure rose to 12.4% (National Health Commission, 2018), indicating inadequate progress. Some studies investigated the professional education background of the existing PHWs and confirmed their inadequate knowledge and skills by conducting examinations or using standard patient methods [10, 11]. Residents’ low confidence in the capacity of PHWs has also been reported as a bottleneck for PHWs to act as gatekeepers in the whole delivery system [12, 13]. Regarding motivation, the well-documented literature on the work motivation of China’s PHWs [14–16] confirmed the low motivation of PHWs in rural China, and identified the limited opportunity for personal growth and career advancement as the most demotivating factors. Improving work motivation does not necessarily mean more money input and in many cases, development can be realized through non-financial incentives such as opportunities for education or training to build skills [17–21].

Training has been regarded by managers of health institutions and administrators of health systems as a key measure to address the low capacity and crisis of primary health workforce in different countries. Recent studies on the trainings for PHWs in China have demonstrated that most PHWs in China have high demands for more effective trainings to improve their knowledge and skills [22], and most PHWs are dissatisfied with some aspects of the trainings, especially the lack of opportunities and the irrelevance of the training material to their actual work [23].

In recent years, health system development has prioritized the capacity building for PHWs. Therefore, many policies are formulated, and different kinds of trainings are organized. PHWs in Chinese THs have three major categories of trainings. One category is the formal and regular continuing professional development training. This kind of training targets those health workers with practice licenses (registered physicians, registered assistant physicians, and registered nurses), and finishing these trainings and getting credits are required for continuation of licenses. These trainings aim to help on-the-job health professionals remain current with the development of medical science and include different forms, including courses, self-study, and writing papers. Another kind of in-service training is organized by health administration departments and different levels of health care institutions (hospitals, centers for disease control, or THs per se) for all types of health workers. These training activities are not organized regularly and aim to improve knowledge or skills in a specific professional discipline based on the institutional development needs or practical policy implementation needs. As some new health programs were added to THs and some traditional functions needed to be strengthened after health system reforms, these kinds of trainings have significantly increased in recent years. For example, “Equalization of Basic Public Health Program,” a key component of health system reform in China, aims to provide a package of basic public health services (14 categories of services, such as diabetes patient management) [24] to residents for free. By improving the capacity of PHWs of THs in providing diabetes patient management care, physicians can be trained to a higher level than hospitals to learn some new skills in the treatment of diabetes patients. Public health workers can also attend a training course introducing the guidelines of diabetes patient management. In addition, General Practitioner (GP) Transfer Trainings are increasing in recent years, aiming to increase the number of GPs in THs. This program tries to transfer current specialists working in THs to GPs by providing some lecturing and practice trainings [25, 26].

We assume that trainings organized by the above efforts could improve the performance in two ways. First, for PHWs who did not receive adequate formal education, medical training programs that were put in place should improve their competence [27]. Second, as a major non-financial incentive, training can stimulate PHWs’ motivation and increase their willingness to make efforts to improve the performance [18, 28–30]. This study employed a mixed method design to draw upon both quantitative and qualitative data to investigate if and how the current training has impacted the PHWs’ competence and work satisfaction with a view to making policy recommendations concerning the training arrangements for PHWs.

## Method

### Study design and sample

We conducted a cross-sectional study in three counties of Shandong province. Located in eastern China, Shandong is the second most populous province and the third largest provincial economy with a per capita GDP per year of ¥68,049 ($10,078 USD). It is also home to the largest number of both registered physicians and PHWs in China [31]. The three counties, Shouguang, Huantai and Yanggu, were randomly selected to represent the province’s high-, middle- and low-level economic region. All the THs in these three counties were included, with 16 from Shouguang, 13 from Huantai, and 18 from Yanggu. All the PHWs on duty on the investigation day were informed and encouraged to participate in the survey. A mixed method research was adopted to describe and assess PHWs’ experiences and perceptions of training.

### Data collection

The research team received specific training for this project and visited to the selected THs to collect data. This research collected both qualitative and quantitative data at the same phase of the project [32, 33].

Quantitative data were obtained from a structured questionnaire which focused on aspects such as socio-demographic characteristics, in-service training, job satisfaction, and a knowledge test. A total of 1147 participants completed the questionnaire, including 590 physicians, 207 nurses, and 351 public health workers.

Qualitative data primarily comprised of a series of semi-structured interviews, which were conducted to better understand the training for PHWs and its impacts. A semi-structured interview guide was designed to elicit respondents’ opinions regarding training, with the main themes focusing on: (1) the views on the content, process and effectiveness of the training, and (2) satisfaction with the training and the impact of training on work motivation. The interviewees were purposefully chosen in consideration of their gender, age, job type, and professional status to make the opinions representative of all PHWs. A total of 203 participants were recruited for the interview including 45 directors/vice-directors of THs, 48 physicians, 43 nurses, and 67 public health workers.

### Measures and variables

The questionnaire used in this study was developed for this study based on primary health system setting in China, and has been translated and listed in the supplementary file.

#### Training

THs in China have dual functions in providing the rural population with both basic medical services (such as clinical treatment for diseases) and public health services (including preventive care, health management services, and health information collection and management). Thus, THs requires multidisciplinary professional teams which consist of physicians, nurses, public health providers, and other health workers. There were differences in the training categories and content they accepted, as introduced above.

In this study, the in-service trainings are measured and analyzed based the length of training: long-term training which lasts 3 to 6 months and short-term training which usually lasts less than 3 days. The long-term training is usually learning by practicing in advanced hospitals and under the supervision of experienced health professionals. The short-term training takes the forms of lectures, workshops, or e-learning [34, 35]. There are two reasons for using the length of trainings as major criteria to differentiate the influences of training. Firstly, the training content differs for various kinds of health workers, while the length of training is a comparable variable for different kinds of health workers. Additionally, the length of training can reflect the forms of trainings (practice or lecturing), determine the influences of training on routine work (leaving a work position or not) and the training gains (practicing skills or accepting knowledge).

#### Competence

Work competence is defined as the knowledge, skills, and ability to successfully perform corresponding work content [36]. In this study, competence was measured through an examination which consisted of questions selected from the Health Professional Licensing Examination (an examination for registered physicians, registered assistant physicians, and registered nurses). A total of three sets of knowledge tests were designed for physicians, nurses, and public health workers respectively, and the assessment was in the form of multiple-choice questions. The test content was based on both general medical knowledge and primary care related knowledge. PHWs who got more than half of the questions wrong were considered incompetent.

#### General job satisfaction

General job satisfaction of PHWs in this study was measured by their responses to the question “Are you satisfied with your work?” The responses of “very satisfied” and “satisfied” were coded as satisfied, while the responses of “moderately,” “dissatisfied,” and “very dissatisfied” were coded as dissatisfied.

A number of covariates were also collected through the questionnaire, including the socio-demographic and work-related characteristics and satisfaction factors. Socio-demographic variables included age (< 30, 30–39, 40–49, ≥50), gender (female, male), and educational background (high school or below, junior college, bachelor and above). Work-related characteristics included professional status (no title, primary, intermediate, senior/deputy senior), employment mode (formal, casual), monthly income (< 3000, 3000–3999, 4000–4999, ≥5000 RMB Yuan), length of service (< 5, 5–9, 10–19, ≥20 years), and professional license for practicing (yes, no). Based on Porter, Cook and Wall’s intrinsic and extrinsic model [37] and empirical research [28, 38], satisfaction factors included intrinsic satisfaction factors (fellow, patient, ability, career, training, participation) and extrinsic satisfaction factors (workplace, income, benefit, performance appraisal, administration, workload). It should be noted that we classified PHWs into physicians, nurses, and public health workers according to the department where they were working, the actual work content they managed, and the type of tests they chose to answer.

### Data analysis

Descriptive statistics were used to describe the social-demographic and work-related characteristics of the PHWs, and Chi-square tests were conducted to determine the differences among different job types of PHWs. Logistic regression was used to identify the association between training and competence and the association between training and job satisfaction for different types of PHWs separately, estimated in terms of the ORs and the corresponding 95% confidence intervals (CIs). Lastly, Kruskal-Wallis rank test was performed to analyze the strength of the association between the general job satisfaction and training, the other satisfaction factors, social-demographic and work-related characteristics. Then, variables associated with general job satisfaction were put into the multiple logistic regression model to further verify whether training influences job satisfaction. All comparisons were two-sided and were considered statistically significant as *p* < 0.05. The quantitative data were analyzed using Stata 14 and missing data were omitted.

Interviews were recorded, transcribed, and analyzed using NVivo12. The researchers first read the interview transcripts to get a sense of the materials as a whole. In the first stage of analysis, open coding was performed to identify the sentences or phrases that contained information relevant to the research questions. In the second stage, these codes were grouped into themes according to their relationships, similarities, and differences. In the third stage, researchers carried out systematic analysis to find the connections of these themes according to the aim of the study [39, 40]. The analysis process was generally inductive: themes emerged from the data which were recorded, coded and classified. The themes and codes were continuously revised, refined and integrated to provide new insights [41].

Analysis results of quantitative data and qualitative data were merged and mutually explained [32, 33], including if and how the training influenced the competency of primary health workers, if and how the training influenced the motivation of primary health workers, if and how the influences were different for different cadres, and if and how different kinds of trainings had different influences.

## Result

### Demographic and work-related characteristics of the participants

Table 1 presents the demographic characteristics of the 1148 PHWs who completed the questionnaire survey. The mean age of the PHWs was 37.89 years (SD = ±8.28). Among them, the mean age of physicians was 38.56 (SD = ±8.35), for nurses it was 35.60 (SD = ±7.04) and among public health workers it was 38.11 (SD ± 8.60). The majority of PHWs were female (64.72%), and PHWs with junior college degrees and primary technical titles represented the largest groups, constituting 41.94 and 44.26% respectively. More than 70% of PHWs had a monthly income of 3000–5000 CNY. Nearly 31.58% of PHWs had worked more than 20 years in present THs, while 21.17% worked less than 5 years. Most PHWs were under a formal employment contract (76.11%) with licenses for practicing (84.49%). There were significant differences in the characteristics among three job types of the PHWs. Table 2 presents the demographic characteristics of the 203 interviewees.

**Table 1:** Demographic and work-related characteristic of participants (N, %)

Characteristics	Overall (*n* = 1148)	Physicians (*n* = 590)	Nurses (*n* = 207)	Public health workers (*n* = 351)	*p* value
Gender					
Male	404 (35.28)	280 (47.62)	4 (1.94)	120 (34.19)	< 0.001
Female	741 (64.72)	308 (52.38)	202 (98.06)	231 (65.81)	
Age					
< 30	185 (16.23)	80 (13.63)	43 (20.87)	62 (17.87)	< 0.001
30–39	472 (41.4)	237 (40.37)	105 (50.97)	130 (37.46)	
40–49	398 (34.91)	223 (37.99)	53 (25.73)	122 (35.16)	
≥ 50	85 (7.46)	47 (8.01)	5 (2.43)	33 (9.51)	
Educational background					
High school or below	228 (19.88)	92 (15.62)	30 (14.49)	106 (30.2)	< 0.001
Junior college	481 (41.94)	222 (37.69)	102 (49.28)	157 (44.73)	
Bachelor and above	438 (38.19)	275 (46.69)	75 (36.23)	88 (25.07)	
Professional status					
No title	220 (19.28)	76 (12.95)	32 (15.53)	112 (32.18)	< 0.001
Primary	505 (44.26)	264 (44.97)	95 (46.12)	146 (41.95)	
Intermediate	398 (34.88)	232 (39.52)	79 (38.35)	87 (25)	
Senior/deputy senior	18 (1.58)	15 (2.56)	0 (0.00)	3 (0.86)	
Employment mode					
Formal	873 (76.11)	490 (83.19)	137 (66.18)	246 (70.09)	< 0.001
Casual	274 (23.89)	99 (16.81)	70 (33.82)	105 (29.91)	
Monthly income (CNY)					
< 3000	285 (24.83)	104 (17.63)	46 (22.22)	135 (38.46)	< 0.001
3000–3999	437 (38.07)	236 (40)	78 (37.68)	123 (35.04)	
4000–4999	426 (37.11)	250 (42.37)	83 (40.1)	93 (26.5)	
≥ 5000	105 (9.13)	69 (9.79)	36 (8.09)		
Length of service (years)					
< 5	238 (21.17)	92 (15.86)	47 (23.15)	99 (29.03)	< 0.001
5–9	200 (17.79)	113 (19.48)	32 (15.76)	55 (16.13)	
10–19	331 (29.45)	184 (31.72)	59 (29.06)	88 (25.81)	
≥ 20	355 (31.58)	191 (32.93)	65 (32.02)	99 (29.03)	
Qualification for practicing					
Yes	964 (84.49)	530 (90.14)	201 (97.57)	233 (67.15)	< 0.001
No	177 (15.51)	58 (9.86)	5 (2.43)	114 (32.85)

**Table 2:** Demographic characteristics of interviewees (N, %)

Characteristics	Overall (*n* = 203)	Directors (*n* = 45)	Physicians (*n* = 48)	Nurses (*n* = 43)	Public health workers (*n* = 67)	*p* value
Gender						
Male	104 (51.23)	42 (93.33)	28 (58.33)	0 (0.00)	34 (50.75)	< 0.001
Female	99 (48.77)	3 (6.67)	20 (41.67)	43 (100.00)	33 (49.25)	
Age						
< 30	31 (44.93)	0 (0.00)	7 (43.75)	12 (57.14)	12 (54.55)	0.097
30–39	19 (27.54)	4 (40.00)	5 (31.25)	6 (28.57)	4 (18.18)	
40–49	18 (26.09)	6 (60.00)	4 (25.00)	3 (14.29)	5 (22.73)	
≥ 50	1 (1.45)	0 (0.00)	0 (0.00)	0 (0.00)	1 (4.55)	
Length of service (years)						
< 5	17 (15.60)	0 (0.00)	2 (8.33)	8 (26.67)	7 (20.59)	0.152
5–9	22 (20.18)	3 (14.29)	5 (20.83)	5 (16.67)	9 (26.47)	
10–19	40 (36.70)	12 (57.14)	11 (45.83)	9 (30.00)	8 (23.53)	
≥ 20	30 (27.52)	6 (28.57)	6 (25.00)	8 (26.67)	10 (29.41)	

**Fig. 1:**
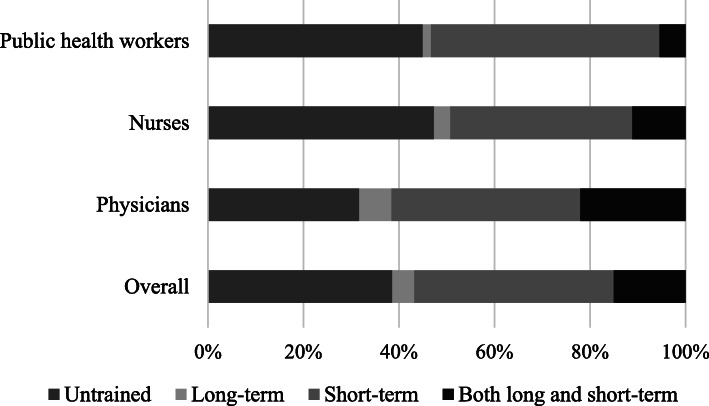
Proportion of training for different job types of PHWs

In general, 443 of 1148 PHWs (38.59%) from our survey had neither received long-term training in the past 3 years, nor taken any short-term training courses in the last year. A comparison of the characteristics of the trained and untrained participants revealed that the proportion of untrained personnel was relatively higher among nurses (98, 47.34%) and public health workers (158, 45.01%) than among physicians (187, 31.69%). All kinds of PHWs mainly received short-term trainings, but the proportion of physicians who received long-term training and both long- and short-term training was higher than that of nurses and public health workers (Fig. 1).

Figure 1 shows the proportion of training for overall PHWs(*n* = 1148), including physicians (*n* = 590), nurses (*n* = 207) and public health workers (*n* = 351).

### Association between training and competence

Table 3 shows the results of the logistic regression and illustrates the associations between the competence of PHWs and training. In general, compared with those without any training, PHWs who received short-term training (OR = 1.619, *p* < 0.01) and who received both long-term and short-term training (OR = 1.589, *p* < 0.05) had significantly higher odds of competence. In the case of physicians, the OR of the competence for those who received long-term training and for those who received both long-term and short-term training were 3.600 (*p <* 0.01) and 2.400 (*p <* 0.01) respectively. The competence of the nurses was neither significantly associated with participation in training nor related to the training type. The public health workers who participated in short-term training had odds of competence 71.0% higher than that of their untrained peers (OR = 1.710, *p* < 0.05).

**Table 3:** Association between training and competence of PHWs

	β	OR	Std. Error	95% CI	*P*
**Overall**					
Training type (reference: untrained)					
Long-term	0.437	1.548	0.451	[0.874,2.74]	0.134
Short-term	0.482	1.619	0.215	[1.248,2.101]	0.000
Both	0.463	1.589	0.287	[1.115,2.264]	0.010
**Physicians**					
Training type (reference: untrained)					
Long-term	1.281	3.600	1.298	[1.776,7.297]	0.000
Short-term	0.590	1.805	0.375	[1.2,2.713]	0.005
Both	0.875	2.400	0.571	[1.506,3.825]	0.000
**Nurses**					
Training type (reference: untrained)					
Long-term	−1.039	0.354	0.305	[0.065,1.912]	0.227
Short-term	0.590	1.803	0.565	[0.975,3.334]	0.060
Both	0.319	1.376	0.651	[0.545,3.476]	0.500
**Public health workers**					
Training type (reference: untrained)					
Long-term	−0.693	0.500	0.440	[0.089,2.808]	0.431
Short-term	0.536	1.710	0.386	[1.099,2.66]	0.017
Both	0.773	2.167	1.124	[0.784,5.987]	0.136

### How training influenced the competence of PHWs

The interview results showed that training could improve PHWs’ competence as they learned about not only advanced technical methods and concepts, but also the interpretation of health reform policies and evaluation indictors, especially for physicians and public health workers.

#### Learn advanced technology and concepts

Some PHWs believed that training could help them broaden their horizon and keep them informed of the cutting-edge knowledge and technology to handle their routine work, thereby improving their competence and medical service.

*I felt I can think more broadly after training. As I recall, during the training last time, physicians from Qufu or Qingdao introduced their experience in public health performance appraisal, which I found very useful. (Public Health Worker 10322)*

*Training can broaden our mind and improve our competence. Communicating with teachers and classmates greatly deepens our understanding and betters our treatment of diseases. (Physician 30531)*

#### Understand policy and assessment requirements

Training provided opportunities for PHWs, especially public health workers, to learn about the in-depth interpretations of the “Equalization of Basic Public Health Services” policy so that they can better understand the policy objectives, work guidelines and assessment criteria for the services package.

*Before the training, we did not have a thorough grasp and understanding of some policies. Thanks to the training and the teacher's explanation, our understanding of the policies deepened. (Public Health Worker 10121)*

*When it comes to public health assessment, the assessment standards vary across counties, provinces, and cities. Last time, an instructor who was a member of the national performance assessment group came to deliver unified training about national public health services. The standards introduced in the provincial level training were about how to fill in the assessment form correctly, but that’s not the case for the national standards which actually focused more on the real work, such as the standards of physical examinations for the elderly. (Public Health Worker 11122)*

In spite of these, the logistic regression results showed that training did not have a significant association with the competence of some types of PHWs, which was related to some problems of training arrangements potentially having negative effects.

#### Failure to address the demands

The main concern of health workers was the mismatch between the training content and the PHWs’ demands. The training content was not based on the actual demand and the clinical nursing skills taught in the training were not applicable in primary healthcare institutions. Meanwhile, some public health workers thought that the training focused more on how to meet the performance assessment criteria of the health administration department instead of the knowledge and skills related to public health services provision such as chronic disease management.

*The training was concerned with some advanced nursing technologies for complex diseases, which, however, were not applicable in primary healthcare institutions. To be honest, in a township hospital, nursing is mainly about injections and a very limited number of treatment care. (Nurse 21141)*

*Some of the trainings I have completed were talking about the same thing, and sometimes the training lacks useful content. (Public Health Worker 10921)*

#### The training being too short

Another complaint about training was the short duration. Even the physicians who received long-term training also considered the training courses not long enough to allow for sufficient practice. The nurses and public health workers who mainly received short-term training had more complaints, and hoped to take part in long-term training that incorporated knowledge, skill, and hands-on practices.

*Short-time training makes little sense, because the instructors talk very fast, making it hard to understand and digest the content. When training time is not enough for effective communication, many things cannot be understood. (Nurse 20841)*

#### Shortage of qualified trainers

Furthermore, some interviewees demanded more professional trainers whose ability would determine the effectiveness of training.

*Our training is basically organized by the county. Trainings organized by the municipal and provincial health department are few and far between. Once, the director of Nursing Department of Peking University Hospital was invited to give a lecture and it worked very well. I think it would be good to invite more experts like this to the primary health institution. (Nurse 21241)*

### Association between training and general job satisfaction

The results of the logistic regression revealed that, compared with those without any training, PHWs who received short-term training (OR = 1.825, *p* < 0.01) and who received both long-term and short-term training (OR = 1.937, *p* < 0.05) had significantly higher odds of general job satisfaction. For physicians, those who received short-term training (OR = 1.916, *p* < 0.01) and who received both types of training (OR = 1.941, *p* < 0.05) had greater odds of general job satisfaction. Regarding nurses, the OR of general job satisfaction for those who received short-term training was 2.697 (*p* < 0.01) compared with their untrained peers. However, general job satisfaction of public health workers was not significantly associated with training. (Table 4).

**Table 4:** Association between training and general job satisfaction of PHWs

	β	OR	Std. Error	95% CI	*P*
**Overall**					
Training type (reference: untrained)					
Long-term	0.460	1.585	0.562	[0.791,3.176]	0.194
Short-term	0.602	1.825	0.289	[1.338,2.490]	0.000
Both	0.661	1.937	0.440	[1.241,3.023]	0.004
**Physicians**					
Training type (reference: untrained)					
Long-term	0.634	1.884	0.804	[0.817,4.349]	0.138
Short-term	0.650	1.916	0.443	[1.217,3.014]	0.005
Both	0.663	1.941	0.533	[1.133,3.326]	0.016
**Nurses**					
Training type (reference: untrained)					
Long-term	−0.345	0.708	0.561	[0.150,3.350]	0.664
Short-term	0.992	2.697	0.999	[1.305,5.573]	0.007
Both	0.926	2.523	1.488	[0.795,8.015]	0.116
**Public health workers**					
Training type (reference: untrained)					
Long-term	0.000	1.000	(empty)		
Short-term	0.314	1.369	0.379	[0.796,2.354]	0.257
Both	1.678	5.355	5.595	[0.691,41.507]	0.108

The multiple logistic regression was conducted to explore how the relevant factors of training influenced the general job satisfaction of PHWs. Variables were put in the logistic regression model in the order of the strength of their association with the outcome in the Kruskal-Wallis equality-of-populations rank test analysis. Almost all socio-demographic and work-related characteristics were taken out of the model due to no significant contribution to the general job satisfaction of PHWs. Finally, the multiple logistic regression analysis revealed that training was significantly associated with general job satisfaction, namely, those who received training were more satisfied than their untrained colleagues (OR = 1.638, *p* < 0.01). (Table 5).

**Table 5:** Determinants of the general job satisfaction by multiple logistic regression analysis

	*β*	OR	Std. Error	95% CI	*P*
Training	0.493	1.638	0.300	[1.144,2.345]	0.007
Satisfaction on income	0.832	2.297	0.763	[1.198,4.403]	0.012
Satisfaction on performance appraisal system	0.188	1.207	0.298	[0.744,1.958]	0.446
Satisfaction on working environment	0.353	1.424	0.288	[0.958,2.117]	0.081
Satisfaction on training	−0.131	0.877	0.177	[0.591,1.303]	0.517
Satisfaction on title promotion	−0.277	0.758	0.219	[0.431,1.334]	0.337
Satisfaction on position promotion	1.145	3.142	0.967	[1.718,5.745]	0.000
Satisfaction on relationship with patients	0.276	1.317	0.274	[0.877,1.979]	0.184
Satisfaction on autonomy	0.853	2.346	0.643	[1.371,4.013]	0.002
Satisfaction on decision-making	0.169	1.184	0.341	[0.673,2.081]	0.558
Satisfaction on workload	0.876	2.400	0.530	[1.557,3.701]	0.000

### How training influenced PHWs’ motivation

The logistic regression results illustrated that training was one of the main factors affecting PHWs’ satisfaction. The interview results revealed that in THs, training not only directly impacted PHWs’ motivation, but it also affected motivation through competence improvement, increase in economic incentives, and promotion prospects.

#### Direct influence

For most THs, training was considered a non-financial reward due to the severe shortage of PHWs and the lack of training opportunities. Therefore, PHWs who had training opportunities felt recognized and appreciated, which resulted in greater motivation. In particular, PHWs with learning-oriented values were more willing to participate in training and were more likely to be motivated by training opportunities. However, too much repetitive and low-quality training that could not satisfy the needs and expectation of PHWs reduced their motivation and disturbed PHWs’ routine work.

*I really like to go to the training, because we must first enrich ourselves to do a better job of leading. I cherish this opportunity to learn. It is a sign of recognition. (Public Health Worker 31021)*

*I don't think it's very useful and the training effect is not satisfactory. You can learn the knowledge from books, and in most cases, the training content is remote from reality of work. (Public Health Worker 20721)*

#### Indirect influence

Training improved PHWs’ motivation through competence improvement, potential influence on economic income, and the chance of promotion.

High-quality training can improve PHW competence, and this personal growth can serve as a reward to the PHWs who can in turn demonstrate stronger work motivation.

*I want to attend training because technologies of treatment have changed a lot. Sometimes, only after learning and practicing can we deepen our understanding. It is necessary to go out to study, and I can do a better job after training. (Physician 20931)*

The association between training and income influenced PHWs’ motivation. PHWs believed that training could help improve their professional competence and then get more patient recognition, thus attracting more patient visits and higher personal income. But some PHWs held a different view, claiming that the absence due to training would cause a reduction in salary or bonus.

*Training should be encouraged as it helps improve personal skills, and it can also increase the income of hospitals and departments. (Physician 10631)*

*I don't want to participate in training as it affects my workload and performance. Additionally, during the training I only get a basic salary. (Physician 30231)*

Some PHWs mentioned that in some institutions, the PHWs that received training were more likely to get professional title promotions or be promoted to managerial positions.

*We are very active in training because it is linked to title promotion. Training is a basic requirement for title promotion, and it is not acceptable if you do not want to participate. (Public Health Worker 20621)*

**Fig. 2:**
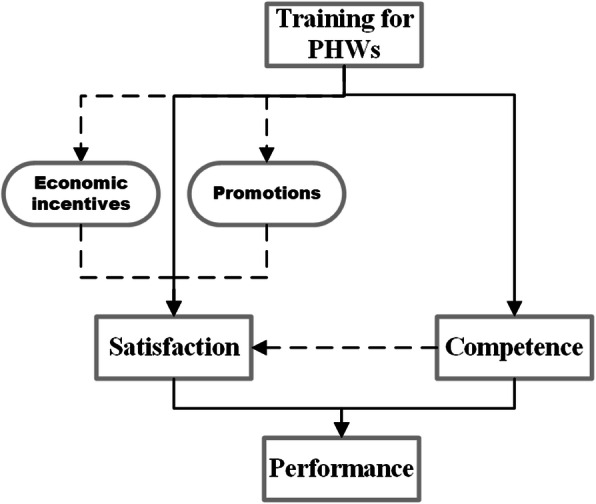
Theoretical framework of how training influenced PHWs’ performance

Figure 2 summarizes the major recurrent themes that emerged from the semi-structured interviews, shedding light upon how training influenced the competence and motivation of PHWs in THs. PHWs can benefit from high-quality training through the acquisition of advanced knowledge and skill as well as a deeper understanding of policy requirements on performance, thus building their competence. However, training may fail its aims if training is unable to address the work needs, training time was limited, or if trainers were not qualified. Work motivation can be evaluated as work satisfaction. Training impacted motivations both directly and indirectly. To a great extent, obtaining training opportunity was considered a recognition for PHWs, and a vital non-financial incentive to improve motivation. But low-quality training that could not address needs and expectation of PHWs could not deliver a positive motivating effect. It was also found that that associations between training with competence improvement, more promotion chances, and higher income could also explain how training impacted PHWs’ satisfaction.

Figure 2 sheds light upon how training influenced the competence and motivation of PHWs in THs.

## Discussion

The PHC institutions in China are responsible for the provision of basic clinical services and public health services to the residents, such as health education, disease prevention, outpatient treatment, non-communicable disease management, and rehabilitation services. Considering the limited number of the in-service PHWs who have received sufficient professional medical education, regular in-service training is crucial to increasing and updating their knowledge. In response, the Chinese government has formulated policies, taken active strategies, and provided financial resources to support the education and training for the purpose of retaining doctors in primary care institutions [8] and increasing accessibility of residents to qualified primary health care [11].

This study collected data from three counties of China’s Shandong province to explore if and how the existing training arrangements influenced work competence and work satisfaction of PHWs in THs. It was found that training was associated with the competence of physicians and public health workers. Both types of trainings had a positive impact on competence of physicians, while short-term trainings were more effective for public health workers. Training had been observed to be one of the main factors affecting general job satisfaction; PHWs who had participated in the training were more satisfied with their job than those who had not.

Both quantitative and qualitative data pointed to the fact that different characteristics of in-service training would influence PHWs in different ways. A review of the existing relevant studies shows that no consensus has been reached as to the association between training and competence, with some studies not finding association between the training received and skill scores [42] and the majority of studies demonstrating that a short-term training course was effective in improving knowledge in the short term [43]. Our analysis results revealed that training with different content, duration and quality had different influences on the competence of different types of PHWs.

According to our results, for different types of PHWs, trainings varied in content and form. Compared with nurses and public health workers, physicians were more likely to participate in long-term training. A larger proportion of physicians (40, 6.78%) received long-term training, and the physicians who participated in long-term training scored higher in the knowledge test than those untrained. One reason of having a higher score is that training content for physicians focused on the latest clinical knowledge and skills related to their specialty. Another reason that training for physicians has greatly increased in recent years is because China launched the health system reform to promote the development of PHC. With this reform, a growing number of professionals originally educated as specialists in THs were trained and transformed to the role of GPs [44]. Public health workers and nurses mainly participated in short-term training. In terms of public health workers, the training materials were related to the provision of basic public health services, including health record management, health education, and health management for the elderly and chronic disease patients [45, 46]. These short-term training sessions were mainly related the implementation of the basic public health services package [24]. For the nurses, the training contents mainly focused on clinical nursing skill and the knowledge related to public health services. However, the results revealed that training failed to improve the competence of nurses. Meanwhile, it was also found that compared with other types of PHWs, a larger proportion of nurses thought that training could not meet their needs and complained about the limited duration of training, which could explain why training did not improve their competence. Another study on the training for nurses had similar results: registered nursing assistants with longer training (more than 75 h) were more likely to report that their training was of high quality and conducive to improving job satisfaction [47].

Qualitative interviews also confirmed the influences of different training arrangements. PHWs can acquire advanced knowledge and skills as well as understand the assessment requirements by taking part in high-quality training. Conversely, training fails to improve PHWs’ competence if the content cannot address the work needs, the duration is short, or the training is delivered by unqualified teachers.

This study also shed light upon the influence of training on PHWs’ motivation, which was measured by general job satisfaction. We found that training was significantly associated to general job satisfaction of PHWs. The general job satisfaction of those who participated in the training was higher than those who did not. We concluded from qualitative research that training not only directly influenced general job satisfaction, but also indirectly improved it by increasing competence, expanding opportunities of higher economic incentives and professional promotion. Training itself was accepted as an important non-financial incentive, and PHWs can have higher satisfaction as a result of obtaining the training opportunity, which they regarded as a kind of recognition [48]. Nonetheless, training did not always have a positive impact on general job satisfaction. PHWs showed disappointment and dissatisfaction when they had to sacrifice working time for training which failed to meet their needs. For example, some PHWs complained that many trainings only introduced assessment criteria applied by supervisors, such as how to fill out assessment forms, which were of limited value in improving their competence or job satisfaction. In addition to the above direct influence, training helped acquire knowledge, which also satisfy the needs of PHWs in personal growth in professional competence. Previous research also showed that knowledge and skills gained through training increased confidence in providing primary healthcare services and job satisfaction [49]. Second, in-service training may be related to general job satisfaction by influencing income, because training may lead to higher pay in the long run. However, completing long-term training means sacrificing their working time, likely also sacrificing some income. In this sense, short-term training could improve competence without sacrificing the current income, thus being more likely to meet PHWs’ expectation. Another influencing channel of training was that PHWs could be motivated by in-service training since it positively correlated with promotion prospects.

Several limitations of our study should be considered. First, it was a cross-sectional study, and the competence and satisfaction before training was not investigated, so the interpretation of causal inference for the results was limited. Additionally, as only three counties from one province were sampled, the national representativeness of the study sample cannot be ascertained.

## Conclusions

In conclusion, the data from the three counties of China’s Shandong Province found that in-service training was associated with an increased knowledge and higher job satisfaction among most PHWs. Qualitative data found that the combination of competence improvement obtained from training and training-related incentives was the reason why training can influence work motivation. Training promoted the competence and satisfaction of PHWs, so as to contribute to the goal of improving performance of health workers. Enhancing the PHWs’ performance and attracting more qualified health workers to primary healthcare institutions is a big challenge for improving the primary health system in most LMICs. This study uses evidence from China’s three rural counties and proposes the necessity of increasing the training opportunities and focusing on the quality of training. Health administrators should make sure that PHWs have access to high-quality training that addresses their work needs. Training could play an important role in motivating PHWs if incentives are in place to link the trainings gains with opportunities for income growth and promotion.

## Supplementary Information

**Additional file 1.**

## Data Availability

The data used and/or analyzed during the study are available from the corresponding author on reasonable request.

## Abbreviations

PHC: Primary health care
SDGs: Sustainable development goals
THs: Township hospitals
PHWs: Primary healthcare workers
LMICs: Low and Middle Income Countries
ORs: Odds ratios
CIs: Confidence intervals
