# Supplementary material for: Training primary healthcare workers in China’s township hospitals: a mixed methods study
Source: BMC Fam Pract. 2020 Dec 2;21:249. doi: 10.1186/s12875-020-01333-4 (PMC7713157; doi:10.1186/s12875-020-01333-4)
Supplement: Supplementary file 1 — Additional file 1. [file 12875_2020_1333_MOESM1_ESM.docx]

# Part 1 Social-demographic characteristics

**1. Gender**

□ Male

□ Female

**2. Birthday __________（dd,mm,yyyy）**

**3. Educational Background**

□ Primary school or below

□ Secondary school

□ High school

□ Junior college

□ Bachelor and above

**4.Professional Status**

□ No title

□ Primary

□ Intermediate

□ Deputy senior

□ Senior

**5. Employment Mode**

□ Formal

□ Casual

**6. Monthly Income: _____________RMB Yuan.**

**7. Length of Service: _____________Years.**

**8. Professional License for Practicing**

□Yes

□ No

**9. Job types**

□ Physician, and your specialty: □ General practice;

□ Internal medicine;

□ Surgery;

□ Paediatrics;

□ Gynaecology;

□ Obstetrician;

□ Traditional Chinese medicine;

□ Mental health;

□ Others_______

□ Nurse, and your specialty: □ Outpatient nursing;

□ Inpatient nursing;

□ Public health;

□ Others________

□ Public health worker

□ Director

# Part 2 In-service Training

**1. Have you ever received long-term training which lasts more than three months in the past three years?**

□Yes

□ No

**1.1 How many months did your last long-term training last?**

**1.2 What was the content of your last long-term training?**

**2. Have you ever received short-term training which lasts less than three days in the past years?**

□Yes

□ No

**2.1 How many days did your last short-term training last?**

**2.2 What was the content of your last short-term training?**

**3. Did the current in-service training satisfy your work demands?**

□ Always

□ Usually

□ Sometimes

□ Occasionally

□ Rarely

# Part 3 Job Satisfaction

**Using this 5-point scale, indicate how satisfied each of the following statements is in describing you.**

|  | very satisfied | satisfied | moderately | dissatisfied | very dissatisfied |
| --- | --- | --- | --- | --- | --- |
| 1.Are you satisfied with your income? |  |  |  |  |  |
| 2.Are you satisfied with the performance criteria? |  |  |  |  |  |
| 3.Are you satisfied with the feedback mechanism of performance? |  |  |  |  |  |
| 4.Are you satisfied with the selection policy of honors or awards? |  |  |  |  |  |
| 5.Are you satisfied with the system of rewards and punishment? |  |  |  |  |  |
| 6.Are you satisfied with promotion standard of profession title? |  |  |  |  |  |
| 7.Are you satisfied with promotion standard of position? |  |  |  |  |  |
| 8.Are you satisfied with doctor-patient relationship? |  |  |  |  |  |
| 9.Are you satisfied with the relationships with colleagues? |  |  |  |  |  |
| 10.Are you satisfied with the support from other departments? |  |  |  |  |  |
| 11.Are you satisfied with your working environment? |  |  |  |  |  |
| 12.Are you satisfied with your workload? |  |  |  |  |  |
| 14.Are you satisfied with training? |  |  |  |  |  |
| 14.Are you satisfied with autonomy in your job? |  |  |  |  |  |
| 15.Are you satisfied with your participation in decision making? |  |  |  |  |  |
| 16.Are you satisfied with your job? |  |  |  |  |  |

# Part 4 Knowledge Test

**1. What is the treatment principle of hypertension in the elderly? (For physicians)**

□ Take 3-4 kinds of hypertensive drugs together.

□ Take hypertensive drugs together at the beginning of hypertension.

□ Start with a small dose and gradually increase if it doesn't work

□ Take large doses for rapid blood pressure reduction.

□ Don’t take any drugs.

**2. What is the priority drug for the treatment of severe pregnancy induced hypertension syndrome? (For physicians)**

□ Hypertensive drugs

□ Diuretics

□ Antispasmodic

□ Sedative

□ Plasma expander

**3.What kind of anti-hypertensive drugs should not be used in treating hypertension complicated with diabetes? (For physicians)**

□ Hydralazine

□ Guanethidine

□ Metoprolol

□ Compound antihypertension

□ Angiotensin converse enzyme inhibitor

**4. Which of the following statements is the benefits of calculus bovis? (For physicians)**

□ Heat-clearing and detoxifying

□ Antispasmodic

□ Make expectoration easy

□ Inducing resuscitation

□ Treating stranguria

**5. What is the purpose of massaging local skin with 50% alcohol? (For nurses)**

□ Disinfection

□ Lubricate

□ Remove dirt

□ Promote blood circulation

□ Lower the body temperature

**6. What is the priority drug for rescuing the penicillin allergic shock? (For nurses)**

□ Calcium gluconate

□ Calcium chloride

□ Hydrochloride adrenaline

□ Norepinephrine

□ Dopamine

**7. What is the first principles of dietary treatment for diabetics? (For public health workers)**

□ Ensure adequate protein

□ Control the fat intake

□ Provide adequate food fiber

□ Control the total energy

□ Reasonable diet

**8.What is the optimal time to take antihypertensive drugs of prolonged action? (For public health workers)**

□ After Getting Up

□ After breakfast

□ After lunch

□ At 3 o’clock in the afternoon

□ Before sleeping in the evening

**9. How old should be initial vaccinated against diphtheria, tetanus and pertussis (DTP)? (For public health workers)**

□ 3 months

□ 4 months

□ 6 months

□ 1 year

**10. Which of the following drug is the first drug for treatment of tuberculosis? (For public health workers)**

□ Isoniazid

□ Sodium aminosalicylate

□ Protionamide

□ Kanamycin
